# Associations between lower extremity muscle mass and metabolic parameters related to obesity in Japanese obese patients with type 2 diabetes

**DOI:** 10.7717/peerj.942

**Published:** 2015-05-05

**Authors:** Hidetaka Hamasaki, Yu Kawashima, Hiroki Adachi, Sumie Moriyama, Hisayuki Katsuyama, Akahito Sako, Hidekatsu Yanai

**Affiliations:** 1 Department of Internal Medicine, National Center for Global Health and Medicine Kohnodai Hospital, Chiba, Japan; 2 General Internal Medicine, Community Healthcare Studies, Jichi Medical University Graduate School, Tochigi, Japan

**Keywords:** Obesity, Resistance training, Muscle mass, Sarcopenia, Type 2 diabetes

## Abstract

**Background.** Age-related loss of muscle mass (sarcopenia) increases the incidence of obesity in the elderly by reducing physical activity. This sarcopenic obesity may become self-perpetuating, increasing the risks for metabolic syndrome, disability, and mortality. We investigated the associations of two sarcopenic indices, the ratio of lower extremity muscle mass to body weight (L/W ratio) and the ratio of lower extremity muscle mass to upper extremity muscle mass (L/U ratio), with metabolic parameters related to obesity in patients with type 2 diabetes and obesity.

**Methods.** Of 148 inpatients with type 2 diabetes treated between October 2013 and April 2014, we recruited 26 with obesity but no physical disability. Daily physical activity was measured by a triaxial accelerometer during a period of hospitalization, and which was also evaluated by our previously reported non-exercise activity thermogenesis questionnaire. We measured body composition by bioelectrical impedance and investigated the correlations of L/W and L/U ratios with body weight, body mass index (BMI), waist circumference (WC), waist-to-hip ratio (WHR), visceral fat area, subcutaneous fat area, serum lipid profile, and daily physical activity.

**Results.** The L/W ratio was significantly and negatively correlated with BMI, WC, WHR, body fat mass, body fat percentage, subcutaneous fat area, and serum free fatty acid concentration, was positively correlated with daily physical activity: the locomotive non-exercise activity thermogenesis score, but was not correlated with visceral fat area. The L/U ratio was significantly and positively correlated with serum high-density lipoprotein cholesterol.

**Conclusions.** High L/W and L/U ratios, indicative of relatively preserved lower extremity muscle mass, were predictive of improved metabolic parameters related to obesity. Preserved muscle fitness in obesity, especially of the lower extremities, may prevent sarcopenic obesity and lower associated risks for metabolic syndrome and early mortality.

## Background

Excess body weight is a serious public health problem and its prevalence has been increasing steadily since 1980 (Finucane et al., 2011). Recommendations to treat obesity by physical activity have emphasized aerobic exercise rather than resistance training (Donnelly et al., 2003; Haskell et al., 2007) because it has been assumed that aerobic exercise results in greater energy expenditure. However, the maintenance of a large muscle mass by resistance training has also been reported to improve cardiovascular disease (CVD) risk factors such as dyslipidemia and type 2 diabetes (Hurley, Hanson & Sheaff, 2011; Braith & Stewart, 2006; Williams et al., 2007) and a recent meta-analysis suggested that resistance training is effective for improving glycemic control in type 2 diabetes (Strasser, Siebert & Schobersberger, 2010).

Sarcopenia is an age-related condition characterized by loss of skeletal muscle mass and decreased muscle strength (Evans, 1995). The prevalence of obesity increases in elderly people with sarcopenia. Sarcopenia is often associated with obesity. Such combination of muscle mass decline and excess adiposity is defined as sarcopenic obesity, which increases the risks for disability and mortality (Zamboni et al., 2008; Prado et al., 2012). Additionally, diabetic older men have significantly lower muscle strength than those without diabetes (Sayer et al., 2005), suggesting that diabetes is an independent risk factor for sarcopenia.

To prevent sarcopenia, it is important to increase muscle mass and strength by resistance training. In elderly people, fat-free mass, especially muscle mass decreased and unfavorable changes in body composition were observed (Buffa et al., 2011). Therefore, we should also treat obesity considering the age of patients. We hypothesized that reduced muscle mass in the lower extremities would be associated with a more sedentary lifestyle, which could lead to or exacerbate sarcopenia. The aim of this study was to investigate the associations of two indices of sarcopenia of the legs; namely, the ratio of lower extremity muscle mass to body weight (L/W ratio) and the ratio of lower extremity muscle mass to upper extremity muscle mass (L/U ratio), with parameters related to obesity, including body mass index (BMI), waist circumference (WC), waist-to-hip ratio (WHR), visceral fat area, subcutaneous fat area, body fat mass, body fat percentage, and serum lipid profile. We also studied influences of age and sex on such parameters.

## Methods

### Study population

Of 148 patients with type 2 diabetes admitted to our hospital for glycemic control between October 2013 and April 2014, we recruited 26 with BMI over 30.0 kg/m^2^, the World Health Organization (WHO) definition of obesity (WHO, 2000). We excluded patients with physical disability such as osteoarthritis. These obese type 2 diabetes patients consisted of 10 men and 16 women, aged 27 to 76 years old. The study protocol was approved by the Medical Ethics Committee of the National Center for Global Health and Medicine (Reference No. NCGM-G-001562), and all subjects gave written informed consent to use their data in the study. This study was performed in accordance with the Declaration of Helsinki.

### Anthropometric measurements

Height and weight were measured using a rigid stadiometer and calibrated scales (seca 764, seca Co., Ltd, Birmingham, United Kingdom). BMI was calculated as body weight in kilograms divided by the square of body height in meters. WC was measured at the umbilical level at the end of exhalation (Lohman, Roche & Martorell, 1988), and hip circumference was measured at the level of the maximum extension of the buttocks posteriorly in the horizontal plane (Lohman, Roche & Martorell, 1988).

### Body composition analysis

Body composition was analyzed using a bioelectrical impedance analysis device (InBody720; Biospace Co., Ltd, Tokyo, Japan). The method is based on the principle that lean body mass contains higher water and electrolyte content than fat tissue, so these tissues can be distinguished by electrical impedance. Segmental body composition was estimated using a patented 8-point tactile electrode system. The device uses 6 frequencies (1, 5, 50, 250, 500, and 1,000 kHz) and produces 30 impedance values for five body segments: right and left upper extremities, trunk, and right and left lower extremities (Anderson, Erceg & Schroeder, 2012). Previous validation study showed that both fat mass and lean body mass evaluated by this device were highly correlated with which measured by dual-energy X-ray absorptiometry (correlation coefficient = 0.832 and 0.899, respectively) (Faria et al., 2014).

### Abdominal fat tissue measurement

Visceral and subcutaneous fat areas were measured using abdominal computed tomography (CT). This CT method was validated previously (Jensen et al., 1995). Single-slice imaging was performed at the umbilical level in a supine position at the end of exhalation using the Aquilion PRIME™ CT system (Toshiba Medical Systems Co., Ltd, Tochigi, Japan). The imaging conditions were 120 kV, 50 mA, and slice thickness of 5 mm. Visceral and subcutaneous fat areas were calculated using commercial software (Fat Measurement; Toshiba Medical Systems Co., Ltd).

### Blood examination

Venous blood samples were taken after a 12-h overnight fast. Fasting plasma glucose was measured using an enzymatic method (GA-1170; Arkray, Inc., Kyoto, Japan). Hemoglobin A1c (HbA1c) was measured by high-performance liquid chromatography (HA-8180; Arkray, Inc.). Total cholesterol, total triglycerides (TG), and high-density lipoprotein cholesterol (HDL-C) were determined enzymatically using the commercially available kits T-CHOKL (Sysmex, Co, Ltd., Hyogo, Japan), Pureauto S TG-N, and Cholestest N HDL (Sekisui Medical Co., Ltd., Tokyo, Japan), respectively. Low-density lipoprotein cholesterol (LDL-C) was calculated by the Friedewald formula (Friedewald, Levy & Fredrickson, 1972). Free fatty acid concentration (FFA) was measured enzymatically using the NEFA-SS Eiken kit (Eiken Chemical Co., Ltd, Tochigi, Japan).

### Physical activity measurements

Physical activity measurements were performed by the method which we previously reported (Hamasaki et al., 2014). Briefly, daily physical activity was measured using a portable triaxial accelerometer during a period of hospitalization (Active Style Pro HJA-350IT; Omron Co., Ltd, Kyoto, Japan). This device differentiated 11 daily activities with almost 100% accuracy (Oshima et al., 2010). Furthermore, metabolic equivalent values (METs) determined by triaxial accelerometry were strongly correlated with METs calculated from energy expenditure as measured by indirect calorimetry (Oshima et al., 2010; Ohkawara et al., 2011). Activity data were stored minute-by-minute and downloaded to a personal computer for analysis. Basal metabolic rate (BMR) was estimated from the following multiple regression equation: BMR (kcal/day) = [(0.1283 + 0.0481 × IBW (kg) + 0.0234 × height (cm) − 0.0138 × age (years) − 0.5473 × sex coefficient (man: 1, woman: 2)) × 293] (Ganpule et al., 2007). Total energy expenditure (TEE) was calculated by regression equation using the MET values obtained by triaxial accelerometry (Oshima et al., 2010). Physical activity level (PAL) was calculated as PAL = TEE/BMR (FAO/WHO/UNU, 1985).

Daily physical activity was also assessed using our previously reported non-exercise activity thermogenesis (NEAT) questionnaire (Hamasaki et al., 2013). Briefly, the questionnaire consists of 11 items about locomotive activities and 25 items about non-locomotive activities. We evaluated each item on a 3-point scale to quantify levels of daily physical activity and then summed the scores to determine the NEAT score. The validity of the NEAT questionnaire was evaluated by comparing scores with objectively measured physical activity levels under free conditions using triaxial accelerometry.

### Statistical analysis

All statistical analysis was performed using SPSS version 19 (IBM Co., Ltd, Chicago, Illinois, USA). All values are expressed as mean ± standard deviation (SD). Sex differences in physiological and biochemical data were analyzed by the Student’s t-test. Correlations of the L/W ratio, L/U ratio and the ratio of upper extremity muscle mass to body weight (U/W ratio) with age, BMI, WC, WHR, TG, HDL-C, LDL-C, FFA, PAL and the locomotive NEAT score were assessed using Pearson’s correlation coefficient. Multiple regression analysis was performed to test the independent correlations of L/W and L/U ratios with physical and biochemical data. Models were adjusted for age. We divided participants into higher and lower BMI groups by the median BMI (≥35.9 kg/m^2^ and <35.9 kg/m^2^). Differences in age, the L/W, L/U and U/W ratios and the locomotive NEAT score between the higher and lower BMI groups were analyzed by the Student’s t-test. A *p* value less than 0.05 was considered statistically significant.

## Results

The mean age of subjects was 50.7 ± 13.2 years. The mean BMI of subjects was 36.2 ± 4.7 kg/m^2^. Eleven subjects were obese class I, 8 were obese class II, and 7 were obese class III (WHO, 2000). About 70% of the subjects (18/26) were never smokers; however, 2 subjects were current smokers and 6 subjects were ex-smokers. Two patients were untreated, 17 patients were treated with oral hypoglycemic agents and 7 patients were treated with combination of oral hypoglycemic agents and insulin. Height, weight, WHR, skeletal muscle mass, the L/W and L/U ratio were significantly higher in men than in women. However, age, serum HDL-C and body fat percentage were significantly higher in women than in men. These results are showed in Table 1. Age was not significantly correlated with L/W, U/W and L/U ratios. The L/W ratio was negatively correlated with BMI, WC, serum FFA, subcutaneous fat area, body fat mass, and body fat percentage, but was not significantly correlated with PAL. However, L/W ratio was significantly and positively correlated with the locomotive NEAT score. The U/W ratio was positively correlated with WHR and was negatively correlated with serum HDL-C, FFA and body fat percentage. The L/U ratio was negatively correlated with weight, BMI, WC, and WHR and was positively associated with serum HDL-C. These results are summarized in Table 2. After adjustment for age, L/W ratio was negatively associated with BMI (*β* = − 0.53, *p* = 0.006), WC (*β* = − 0.539, *p* = 0.005), serum FFA (*β* = − 0.425, *p* = 0.029), subcutaneous fat area (*β* = − 0.678, *p* < 0.001), body fat mass (*β* = − 0.623, *p* = 0.001) and body fat percentage (*β* = − 0.896, *p* < 0.001), and positively associated with the locomotive NEAT score (*β* = 7.02, *p* < 0.001). After adjustment for age, L/U ratio was also negatively associated with weight (*β* = − 0.441, *p* = 0.036), BMI (*β* = − 0.484, *p* = 0.013), WC (*β* = − 0.499, *p* = 0.011) and WHR (*β* = − 0.727, *p* < 0.001), and positively associated with serum HDL-C (*β* = 0.542, *p* = 0.006). No significant association was found between the L/U ratio and visceral fat area, subcutaneous fat area, body fat mass, or body fat percentage. In men, the L/W ratio was negatively correlated with BMI, WC, subcutaneous fat area, body fat mass, and body fat percentage. However, in women, the L/W ratio was negatively correlated with only body fat percentage and was positively correlated with the locomotive NEAT score. The L/U ratio was negatively correlated with WC and WHR in men. The L/U ratio was also negatively correlated with BMI and WHR in women. Moreover, the L/U ratio was positively correlated with serum HDL-C. Table 3 showed these single correlations of L/W ratio, U/W ratio and L/U ratio with clinical parameters.

**Table 1:** Clinical characteristics in men and women.

	Men	Women	*p* value for sex difference
Age, years	43.6 (12.1)	55.1 (12.1)	0.027
Height, cm	166.2 (8.6)	157.1 (7)	0.007
Weight, kg	101.5 (13.9)	88.5 (13.2)	0.026
BMI, kg/m^2^	36.9 (5.7)	35.8 (4.2)	0.582
Waist circumference, cm	116.6 (13.7)	110.8 (11.1)	0.247
Waist-to-hip ratio	1.05 (0.09)	0.97 (0.07)	0.032
Fasting plasma glucose, mg/dL	123.2 (38.5)	128.8 (35.1)	0.709
HbA1c, %	8.9 (2.3)	7.9 (1.8)	0.225
Total cholesterol, mg/dL	164.1 (27.5)	181.7 (46.2)	0.288
Triglycerides, mg/dL	195 (84.4)	175 (153.4)	0.071
HDL cholesterol, mg/dL	34.4 (7.3)	44.6 (13.6)	0.04
LDL cholesterol, mg/dL	92 (31.2)	102.8 (37.8)	0.458
Free fatty acids, µEq/L	614.9 (190.8)	727.4 (191.2)	0.157
Visceral fat area, cm^2^	253 (87)	258 (121)	0.918
Subcutaneous fat area, cm^2^	380 (156)	371 (96)	0.855
Skeletal muscle mass, kg	32.3 (3.6)	24.8 (4.3)	<0.001
Body fat mass, kg	41.6 (13.3)	41.4 (7.7)	0.971
Body fat percentage, %	40.9 (9.6)	47.5 (3.4)	0.019
L/W ratio	0.18 (0.03)	0.16 (0.02)	0.033
U/W ratio	0.062 (0.01)	0.062 (0.01)	0.911
L/U ratio	2.88 (0.34)	2.57 (0.28)	0.01
Physical activity level	1.45 (0.1)	1.46 (0.1)	0.744
Basal metabolic rate, kcal/day	1561 (392)	1358 (397)	0.221
Locomotive NEAT score	18.5 (3.9)	16.4 (4)	0.193

**Notes.**

Data are expressed as mean (SD).

BMI body mass index HbA1c hemoglobin A1c HDL high-density lipoprotein LDL low-density lipoprotein L/W ratio the ratio of lower extremity muscle to body weight U/W ratio the ratio of upper extremity muscle to body weight L/U ratio the ratio of lower to upper extremity muscle mass NEAT non-exercise activity thermogenesis

**Table 2:** Correlations of L/W ratio, U/W ratio and L/U ratio with physiological and biochemical parameters.

	L/W ratio		U/W ratio		L/U ratio	
	Correlation coefficient	*p* value	Correlation coefficient	*p* value	Correlation coefficient	*p* value
Age, years	−0.233	0.251	−0.348	0.082	0.215	0.29
BMI, kg/m^2^	−0.474	0.014	−0.064	0.756	−0.506	0.008
Waist circumference, cm	−0.458	0.019	−0.013	0.948	−0.523	0.006
Waist-to-hip ratio	−0.18	0.388	0.511	0.009	−0.742	<0.001
Triglycerides, mg/dL	0.152	0.459	−0.209	0.305	0.13	0.528
HDL cholesterol, mg/dL	−0.136	0.507	−0.483	0.012	0.561	0.003
Free fatty acids, µEq/L	−0.526	0.006	−0.391	0.049	0.047	0.821
Visceral fat area, cm^2^	−0.034	0.869	−0.074	0.72	0.05	0.807
Subcutaneous fat area, cm^2^	−0.526	0.006	−0.230	0.259	−0.331	0.099
Body fat mass, kg	−0.571	0.002	−0.261	0.198	−0.327	0.101
Body fat percentage, %	−0.908	<0.001	−0.8	<0.001	−0.007	0.974
PAL	0.129	0.633	−0.055	0.839	0.258	0.335
Locomotive NEAT score	0.634	<0.001	0.387	0.051	0.223	0.273

**Notes.**

L/W ratio the ratio of lower extremity muscle mass to body weight U/W ratio the ratio of upper extremity muscle mass to body weight L/U ratio the ratio of lower to upper extremity muscle HDL high-density lipoprotein LDL low-density lipoprotein PAL physical activity level NEAT non-exercise activity thermogenesis

**Table 3:** Correlations of L/W ratio, U/W ratio and L/U ratio with clinical parameters in men and women.

	Men				Women			
	L/W ratio		L/U ratio		L/W ratio		L/U ratio	
	Correlation coefficient	*p* value	Correlation coefficient	*p* value	Correlation coefficient	*p* value	Correlation coefficient	*p* value
Age, years	0.032	0.931	−0.435	0.208	−0.16	0.553	0.259	0.332
BMI, kg/m^2^	−0.811	0.004	−0.437	0.206	−0.275	0.302	−0.596	0.015
Waist circumference, cm	−0.878	0.001	−0.642	0.046	−0.345	0.191	−0.485	0.057
Waist-to-hip ratio	−0.392	0.297	−0.76	0.017	−0.387	0.138	−0.773	<0.001
Triglycerides, mg/dL	0.024	0.948	0.296	0.407	0.222	0.409	0.139	0.606
HDL cholesterol, mg/dL	−0.147	0.685	−0.386	0.271	0.152	0.574	0.643	0.007
Free fatty acids, µEq/L	−0.529	0.115	−0.123	0.734	−0.189	0.484	−0.024	0.929
Visceral fat area, cm^2^	−0.305	0.391	−0.233	0.517	0.167	0.536	0.106	0.695
Subcutaneous fat area, cm^2^	−0.806	0.005	−0.433	0.211	−0.255	0.34	−0.368	0.161
Body fat mass, kg	−0.925	<0.001	−0.564	0.09	−0.125	0.644	−0.32	0.227
Body fat percentage, %	−0.975	<0.001	−0.412	0.237	−0.726	0.001	−0.101	0.709
PAL	0.651	0.234	0.038	0.951	−0.062	0.855	0.279	0.406
Locomotive NEAT score	0.621	0.055	0.381	0.278	0.618	0.011	0.335	0.205

**Notes.**

L/W ratio the ratio of lower extremity muscle mass to body weight U/W ratio the ratio of upper extremity muscle mass to body weight L/U ratio the ratio of lower to upper extremity muscle HDL high-density lipoprotein LDL low-density lipoprotein PAL physical activity level NEAT non-exercise activity thermogenesis

We also examined the differences in age, sex ratio, L/W, U/W and L/U ratios, and the locomotive NEAT score between the higher BMI group (≥35.9 kg/m^2^) and lower BMI group (<35.9 kg/m^2^). Both L/W and L/U ratios were significantly lower in the higher BMI group than in the lower BMI group. Furthermore, the locomotive NEAT score was also significantly lower in the higher BMI group (Table 4). On the contrary, no differences in age, sex ratio and PAL were observed between the higher and lower BMI groups.

**Table 4:** Comparison of age, sex, L/W ratio, U/W ratio, L/U ratio and locomotive NEAT score between the higher BMI group (BMI ≥35.9) and lower BMI group (BMI <35.9).

	Higher BMI group	Lower BMI group	*p* value
Age, years	48.7 (12.8)	52.7 (13.8)	0.45
Sex (men/women)	6/7	4/9	0.239^a^
L/W ratio	0.16 (0.02)	0.18 (0.03)	0.044
U/W ratio	0.06 (0.01)	0.06 (0.01)	0.911
L/U ratio	2.57 (0.28)	2.88 (0.34)	0.016
Physical activity level	1.47 (0.1)	1.45 (0.1)	0.68
Locomotive NEAT score	15.5 (4)	18.9 (3.4)	0.032

**Notes.**

Data are expressed as mean (SD).

L/W ratio the ratio of lower extremity muscle to body weight U/W ratio the ratio of upper extremity muscle to body weight L/U ratio the ratio of lower to upper extremity muscle mass NEAT non-exercise activity thermogenesis.

a Chi-square test was performed

## Discussion

Numerous studies have suggested that moderate regular aerobic exercise can lead to weight loss, fat mass loss, and improved insulin sensitivity in obesity (McTiernan et al., 2007; Houmard et al., 2004). Resistance training has also been shown effective for improving metabolic risk factors, including insulin resistance and dyslipidemia (Braith & Stewart, 2006); however, the extent to which resistance training can improve metabolic risk factors and the mechanism involved are still unclear. We found that lower extremity muscle mass was a critical determinant of favorable metabolic status in obese type 2 diabetes patients, implying that maintaining leg muscle strength through resistance training may help reduce risk factors for metabolic disease and early mortality.

Several previous studies reported an inverse association between muscle strength and metabolic risks (Steene-Johannessen et al., 2009; Jurca et al., 2005). Obesity increases intramyocellular TG in skeletal muscle, which impairs insulin sensitivity and decreases muscle strength (Amati et al., 2011; Kelley, 2002). Accordingly, resistance training for improving muscle fitness should be performed to prevent or improve metabolic risk factors such as insulin resistance and dyslipidemia.

In the present study, higher L/W and L/U ratios were associated with favorable BMI, WC, WHR, serum FFA, and serum HDL-C. In addition, both L/W and L/U ratios were lower (i.e., lower extremity muscle mass was reduced) in patients with higher BMI. On the other hand, higher U/W ratio was associated with unfavorable WHR and serum HDL-C. These results strongly suggest that not upper extremity but adequate lower extremity muscle mass is critical for reducing metabolic risk factors in obese type 2 diabetes patients.

### Lower extremity muscle mass and age

Sarcopenia is an age-related condition characterized by loss of skeletal muscle mass (Evans, 1995). Lower extremity muscle mass will decrease with age. However, no significant correlations between L/W or L/U ratios and age were observed in this study. After adjustment for age, associations of L/W and L/U ratios with favorable metabolic values remained. Although we cannot always generalize our results to the general population, L/W and L/U ratios may be useful indicators of lower extremity muscle mass in wide range of age groups.

### Lower extremity muscle mass and daily physical activity

Obese patients tend to have a sedentary lifestyle, which increases body weight and decreases muscle mass and strength in the lower extremities, and these in turn contribute to a continued sedentary lifestyle. The locomotive NEAT score was in fact significantly reduced in the higher BMI group, suggesting that the most obese individuals tended not to walk as often as subjects in the lower BMI group. We also observed a significant and positive correlation between the L/W ratio and the locomotive NEAT score, suggesting that increased locomotive activity is associated with increased muscle mass. While we did not find significant correlations of PAL with the L/W and L/U ratios, PAL was measured during a period of hospitalization and so these findings may not represent the true physical activity of subjects in daily life outside the hospital.

### Lower extremity muscle mass and lipid metabolism

Free fatty acid levels are usually elevated in obese patients because the enlarged adipose tissue releases FFA and FFA clearance is reduced (Boden, 2008). Elevated FFA levels cause insulin resistance in skeletal muscle; indeed, high FFA levels are responsible for about 50% of the insulin resistance in obese patients with type 2 diabetes (Boden, 2008). Lowering FFA levels can improve insulin resistance. Leg muscle is the predominant FFA uptake tissue, accounting for 15% to 20% of FFA uptake at rest and 30% to 60% during exercise (Jensen, 2003). Therefore, lower extremity muscle fitness and locomotive activity are essential for maintaining proper FFA metabolism. A significant and negative correlation of the L/W ratio with FFA levels suggests that increasing lower extremity muscle mass is associated with improvement of FFA metabolism and insulin resistance in obese type 2 diabetes patients.

Dyslipidemia in obesity is typically characterized by elevated serum TG and FFA as well as lower HDL-C with HDL dysfunction (Mineo & Shaul, 2012). HDL metabolism is strongly impaired in obesity, which increases CVD risk. However, Stensvold et al. (2010) found no significant association between dynamic resistance training and HDL-C (Stensvold et al., 2010). In contrast, the significant and positive correlation between the L/U ratio and HDL-C in the present study suggests that larger muscle mass of the lower extremities has a favorable effect on lipid metabolism even without a resistance training program. Moreover, higher HDL-C levels are associated with physical performance indices such as walking speed (Landi et al., 2007), indicating that lower extremity muscle fitness has some associations with increase of HDL-C levels. Why this association was found in the present study but not by Stensvold et al. (2010) warrants further study given the strong link between HDL-C and CVD risk.

A systematic review and meta-analysis to investigate the independent and combined effects of aerobic exercise and resistance training on visceral fat area showed that aerobic exercise was effective in reducing visceral fat area, while resistance training was not (Ismail et al., 2012). However, Yagi et al. (2014) found that higher skeletal muscle mass of the lower limbs was associated with decreased visceral fat area in healthy men, again suggesting the importance of locomotive activities for the control of obesity and metabolic syndrome. The exact reason why a negative correlation of L/W ratio with visceral fat area was not observed is unclear. It may be related to that subcutaneous fat obesity is dominant in our study population. Misra et al. (2008) showed that moderate intensity resistance training reduced subcutaneous adipose tissue in patients with type 2 diabetes, which suggests that resistance training can reduce subcutaneous fat but not visceral fat in patients with type 2 diabetes. Indeed, we found significant and negative correlations of the L/W ratio with WC, body fat mass, body fat percentage, and subcutaneous fat area, suggesting that increasing lower extremity muscle mass can lead to a reduction in fat mass.

### Sex differences

In the present study, we observed more significant correlations of L/W and L/U ratios with metabolic parameters related to obesity in men than in women. On the other hand, L/U ratio was positively correlated with serum HDL-C in women not in men. However, the potential mechanism of sex differences is unknown. Further studies are needed to elucidate the differences in the role of lower extremity muscle mass between men and women.

### Limitations

Limitations to our study include the cross-sectional study design, small sample size, and potential confounding factors. The cross-sectional study design does not allow causal relationships to be deduced, and future prospective studies are therefore needed to confirm our results. We recruited obese patients according to WHO definition of obesity, with BMI over 30.0 kg/m^2^; however, obesity was defined as a BMI over 25.0 kg/m^2^ in Japanese population (Examination Committee of Criteria for ‘Obesity Disease’ in Japan; Japan Society for the Study of Obesity, 2002). We should include such overweight patients in a future study. In addition, about 70% of the participants (18/26) were currently taking cholesterol-lowering agents such as statins and fibrates, which are known to increase HDL-C levels. Third, we did not evaluate muscle strength of the lower extremities. However, a positive correlation between muscle mass and muscle strength is well established (Janssen et al., 2000; Newman et al., 2003; Chen et al., 2013). Forth, PAL was measured during a period of hospitalization and so these findings may not represent the true physical activity of subjects in daily life outside the hospital.

## Conclusions

We demonstrated that lower extremity muscle mass is predictive of reduced body weight, BMI, WC, WHR, subcutaneous fat area, body fat mass, body fat percentage, and serum FFA as well as higher serum HDL-C in obese patients with type 2 diabetes. Our results suggest that lower extremity muscle mass is more important than upper extremity muscle mass for improving metabolic risk factors. Resistance training targeting the lower extremities may allow obese patients to increase physical activity, preventing sarcopenic obesity and concomitant risks of morbidity and mortality.

## Supplemental Information

10.7717/peerj.942/supp-1 Supplemental Information 1 Raw data
